# Experimental Immunization Based on *Plasmodium* Antigens Isolated by Antibody Affinity

**DOI:** 10.1155/2015/723946

**Published:** 2015-10-11

**Authors:** Ali N. Kamali, Patricia Marín-García, Isabel G. Azcárate, Antonio Puyet, Amalia Diez, José M. Bautista

**Affiliations:** ^1^Department of Biochemistry and Molecular Biology IV, Universidad Complutense de Madrid, Facultad de Veterinaria, Ciudad Universitaria, 28040 Madrid, Spain; ^2^Department of Biology, Faculty of Basic Sciences, Islamic Azad University, Central Tehran Branch, Tehran 14676-86831, Iran; ^3^Department of Medicine and Surgery, Psychology, Preventive Medicine and Public Health and Medical Immunology and Microbiology, Faculty of Health Sciences, Universidad Rey Juan Carlos, Alcorcón, 28922 Madrid, Spain; ^4^Research Institute Hospital 12 de Octubre, Universidad Complutense de Madrid, 28040 Madrid, Spain

## Abstract

Vaccines blocking malaria parasites in the blood-stage diminish mortality and morbidity caused by the disease. Here, we isolated antigens from total parasite proteins by antibody affinity chromatography to test an immunization against lethal malaria infection in a murine model. We used the sera of malaria self-resistant ICR mice to lethal *Plasmodium yoelii yoelii* 17XL for purification of their IgGs which were subsequently employed to isolate blood-stage parasite antigens that were inoculated to immunize BALB/c mice. The presence of specific antibodies in vaccinated mice serum was studied by immunoblot analysis at different days after vaccination and showed an intensive immune response to a wide range of antigens with molecular weight ranging between 22 and 250 kDa. The humoral response allowed delay of the infection after the inoculation to high lethal doses of *P. yoelii yoelii* 17XL resulting in a partial protection against malaria disease, although final survival was managed in a low proportion of challenged mice. This approach shows the potential to prevent malaria disease with a set of antigens isolated from blood-stage parasites.

## 1. Introduction

Human malaria infection can lead to a wide range of clinical symptoms that are influenced by epidemiological and immunological factors [1] along with the mechanisms of immune evasion of the parasite [2]. Protective humoral response against* Plasmodium falciparum* can be acquired after repeated infections of malaria; however, it does not persist over long periods of time and it is generally incomplete [1].

Despite concerted efforts worldwide, most advanced vaccines in development have shown moderate efficacy [3] maybe since they are based on parasite antigens, too polymorphic, and expressed only in brief periods of the parasite life cycle [4]. In addition, vaccine candidates represent less than 0.5% of the entire genome [5] and more than 50% of the vaccines currently designed are based independently on only three antigens: circumsporozoite protein (CSP), merozoite surface protein (MSP), and the apical membrane antigen 1 (AMA-1). Due to difficulties in identifying the widely dispersed immune responses to* Plasmodium*, only about 1% of the antigens encoded by the parasite have been studied so far [6]. Although the identification of immunological markers for protection has been intricate, several reports suggest that humoral responses to a variety of antigens are involved in protection [5, 7].

Thus, a combination of antigen subunits of different parasite stages is the long-term objective of malaria vaccine [8]. Vaccines based on the inoculation of the whole organism enable a vast array of antigens to be delivered and therefore provide a multiepitope vaccine [9–11]. Nevertheless, the specific antigens mediating protective immunity induced by whole organism vaccination are still largely unknown [11]. The generation of protective responses in malaria induced by subunit vaccines is still under debate [11, 12]. In addition, there may be specific protective immune responses that do not arise during the normal course of infection but that can be primed by vaccination and boosted by infection [12] or by ultralow doses of drug-contained or killed parasites [13–15].

By using animal models, several laboratories have attempted to induce protective immune response by immunization with crude preparations of whole blood-stage antigens applying various adjuvants, and different degrees of protection have been reported [15–17]. Protective response in mice induced by undetermined whole blood-stage antigens has been shown to be dependent on CD4^+^ T cells, interferon gamma, and nitric oxide [15]. Thus, with more than 5,000 proteins expressed during the life cycle of* Plasmodium* spp. [18] it remains to be known which combinations of them could be efficient antigens mediating protective immunity induced by whole organism vaccination. Moreover, a question arising from these studies is whether immune protection is elicited predominantly by a very limited or a large number of antigens [19].

Previous results from our laboratory show that a percentage of ICR mice naturally acquire a long-term protective humoral response against homologue reinfections of the lethal parasite* P. yoelii yoelii* 17XL (*PyL*) [20]. Moreover, sera of these mice are a suitable source of immunoglobulins for the isolation of antigenic plasmodial proteins by immunoaffinity [21]. Consequently, in the present study, we have investigated the use of the protective IgGs from pooled sera of malaria model in ICR mice [20] to isolate blood-stage parasite antigens by immunoaffinity as described earlier [21] to investigate their immunogenicity in BALB/c mice. The immunization elicited antigens and the level of protection achieved against a lethal malaria challenge is discussed.

## 2. Materials and Methods

### 2.1. Animals and Malaria Infection

The rodent malaria parasite* PyL* MRA-267 was obtained from Dr. Virgilio Do Rosario (Instituto de Higiene e Medicina Tropical, Universidade Nova de Lisboa) and stored in liquid nitrogen after serial blood passages in mice. Infected blood was kept in liquid nitrogen in a solution containing glycerol 28% (v/v), sorbitol 3% (w/v), and NaCl 0.65% (w/v). Inbred BALB/cAnNHsd and random-bred ICR pathogen-free female mice (Hsd:ICR[CD-1]), aged 6–8 weeks, were purchased from Harlan Laboratories (Udine, Italy). The mice were housed under standard conditions of light (12 : 12 h light : dark cycles), temperature (22–24°C), and humidity (around 50%) in the Animal Housing Facility at Universidad Complutense de Madrid. All mice were fed a commercial diet (2018 Teklad Global 18% Protein Rodent Diet, Harlan Laboratories)* ad libitum*. All animal care and experimental procedures carried out at the Universidad Complutense de Madrid complied with Spanish (R.D. 32/2007) and European Union legislation (2010/63/CE) and were approved by the Animal Experimentation Committee of this institution. The experiments here described involving animals are reported following the ARRIVE guidelines [22].

For all challenges, parasite isolation, and immune sera sampling, mice were inoculated by intraperitoneal (i.p.) injection of 2 × 10^7^ *PyL*-infected red blood cells (iRBCs) from infected mice. After infection, p-aminobenzoic acid at a final concentration of 0.05% (w/v) was included in the drinking water to enhance parasite growth [23, 24].

### 2.2. Total Parasite Protein Extraction

Protein lysates were extracted from the RBCs with a mixture of ring, trophozoite, and schizont-stage parasites of infected ICR mice showing >50% parasitaemia. Mice were anaesthetized with isoflurane, as recommended by the local Animal Experimentation Committee, and whole blood was collected from the aorta into tubes containing EDTA 0.1 M as anticoagulant and kept at −80°C until protein extraction. Protein isolation began with RBC lysis using 10 vol saponin 0.1% (w/v) in PBS. After centrifugation (320 ×g, 5 min, 4°C) and washing twice in cold PBS, the pellet was treated with extraction buffer (50 mM Tris-HCl, pH 8.0; 50 mM NaCl; 0.5% Mega 10) containing protease inhibitor cocktail (Roche, Indianapolis, IN, USA) and subjected to four freeze-thaw cycles. Finally, lysates were centrifuged (20,000 ×g, 15 min, 4°C) and total* PyL* protein samples stored at −80°C until use.

### 2.3. Purification of Mouse IgGs

Hsd:ICR (CD-1) malaria-resistant mice were generated as previously described by Azcárate et al. [20]. Briefly mice were infected intraperitoneally with 2 × 10^7^ PyL-iRBCs obtained from donor* PyL*-infected mice. Mice that recovered from 1st infection were reinfected on days 60 and 420 after the first infection following the same infection protocol. IgGs from 150 *μ*L of pooled serum samples obtained after three infections of malaria-resistant ICR mice were isolated using 0.2 mL NAb protein A/G column (Thermo Scientific, MA) according to the manufacturer's instructions. Briefly, serum was diluted in binding buffer (sodium phosphate 100 mM containing NaCl 150 mM, pH 7.2) and IgGs bound to the column were eluted in 400 *μ*L fractions using the elution buffer provided (pH 2.8). Collecting tubes were previously preloaded with 40 *μ*L of Tris-HCl 1 M, pH 8.5 for neutralization. After elution, purified IgGs were dialyzed in a Slid-A-Lyzer dialysis cassette (Thermo Scientific) against 500 mL of sodium phosphate 0.01 M containing NaCl 0.15 M, pH 7.2 during 2 h, with a total of 3 replacements. After the third replacement, equilibrium was continued overnight at 4°C and dialyzed IgGs were kept at −20°C until use. Purity and yield of IgGs were verified by sodium dodecyl sulfate-polyacrylamide gel electrophoresis and western blotting. Contaminating protein in the elution and loss of IgGs during purification, dialysis, and concentration were both negligible as previously reported [21].

### 2.4. Immobilization of Mice IgGs

Isolated mice IgGs were immobilized on agarose using the Pierce Direct IP kit (Thermo Scientific, MA), following the procedure previously reported [21]. Briefly, 100 *μ*L of AminoLink Plus coupling resin was applied to each column and centrifuged at 1,000 ×g for 1 min. The columns were then washed twice with 200 *μ*L of 1x coupling buffer (sodium phosphate 0.01 M containing NaCl 0.15 M, pH 7.2). Next, 50 *μ*g of purified IgGs from malaria-resistant mice was loaded onto the column and the volume immediately adjusted to 200 *μ*L using ultrapure water and coupling buffer. After addition of 3 *μ*L of sodium cyanoborohydride 5 M to allow covalent binding, the column was incubated at room temperature with rotation for 2 h. Next, the column was washed twice with coupling buffer and prewashed with 200 *μ*L of quenching buffer (Tris-HCl 1 M) to remove any uncoupled IgG. To block the remaining sites on the resin, 200 *μ*L of quenching buffer and 3 *μ*L of sodium cyanoborohydride were once again added, and the column was incubated for 15 min with gentle shaking. Finally, the column was washed twice with coupling buffer and 6 times with wash solution (NaCl 1 M) and subjected to a final wash with TBS (Tris-buffered saline, Tris 0.025 M, NaCl 0.15 M; pH 7.2).

### 2.5. Isolation of Parasite Antigens by Immunoaffinity

This procedure has been previously reported [21]. Briefly, total proteins extracted from* PyL* (500–1000 *μ*g) diluted in a 600 *μ*L volume of TBS were loaded onto each antibody-coupled spin column and incubated for 2 h with gentle shaking. To remove nonbound proteins, the complex was washed three times with TBS and once with conditioning buffer supplied with the kit. Sodium deoxycholate 1% (w/v) (Sigma-Aldrich, St. Louis, MO, USA) in PBS was used to dissociate the bound antigens from the immobilized antibody and the eluted antigens were recovered in PBS [25]. The eluted antigens were concentrated and buffer was exchanged with PBS using Pierce Concentrator 7 mL/9 K (Thermo Scientific, MA).

Protein concentration in all fractions, from parasite extracts to antigen isolation, was determined with the Bio-Rad DC Protein Assay (Bio-Rad, CA, USA).

### 2.6. Immunoblot Assays

Parasite total protein extracts, prepared as described above, were solubilized in SDS-polyacrylamide gel electrophoresis (SDS-PAGE) sample buffer containing SDS 2.5%, boiled for 5 minutes, and subsequently separated on 10% SDS-PAGE. After electrophoresis, proteins were transferred onto PVDF (Hybond-P, GE Healthcare) membranes following standard procedures. Blots were blocked for 1 h with PBS containing 5% nonfat dried milk. The blots were subsequently incubated overnight with individual mouse serum from any of the experimental groups of immunized mice, diluted in PBS containing 0.05% Tween-20 at concentration of 0.05%. Bound IgGs were detected using HRP-conjugated anti-mouse IgG (GE Healthcare) at 1/5000 dilution. Detection was performed using the SuperSignal chemiluminescence substrate (Thermo Scientific, MA) and exposure to X-ray film.

### 2.7. Adjuvants and Immunizations

To compare CPG ODN-1826 (InvivoGen) and Freund's adjuvants, 60 BALB/c mice were divided into 6 groups (*n* = 10 each) and inoculated with same volume (50 *μ*L) containing 2 *μ*g of the immunoaffinity-purified antigens in different preparations as follows. Group 1: intramuscular (i.m.) injection of one volume of antigens plus 50 *μ*g CpG ODN_1826_ plus one volume of incomplete Freund's adjuvant (IFA). Group 2: i.p. injection of one volume of antigens plus one volume of Complete Freund's Adjuvant (CFA). Group 3: i.p. injection of antigens alone. Group 4: i.m. injection of one volume of 50 *μ*g CpG ODN_1826_ plus one volume of IFA. Group 5: i.p. injection of CFA. Group 6: i.p. injection of PBS. Inoculations were performed twice on days 1 and 27. Four weeks later, at day 55, animals were i.p. infected with 2 × 10^7^ *PyL* parasitized RBCs. Parasitemia was monitored by thin tail-blood smears, stained with Wright's eosin methylene blue. For boosting, CFA (1 : 1 emulsion) was replaced with the IFA (1 : 1 emulsion) in groups 2 and 5.

For the second vaccination trial, immunizations were performed only with the Freund's adjuvant system. At days 1, 25, 50 and 85, three groups of mice were immunized subcutaneously (s.c.) with 10 *μ*g of the immunoaffinity-purified antigenic preparations plus adjuvant (group 1), with only adjuvant (group 2), and with PBS (group 3). CFA was used only in the primary immunization and was replaced with IFA for the following boosts. Two weeks after the last immunization, animals were infected intraperitoneally as indicated above.

### 2.8. Statistical Analysis

Differences between individual groups were analyzed using Student's *t*-test or the Mann-Whitney test in Prism 5 software (GraphPad Software Inc.). Significance was set at *P* < 0.05.

## 3. Results

### 3.1. Diversity of Blood-Stage Antigens Isolated by Immunoaffinity

**Figure 1:**
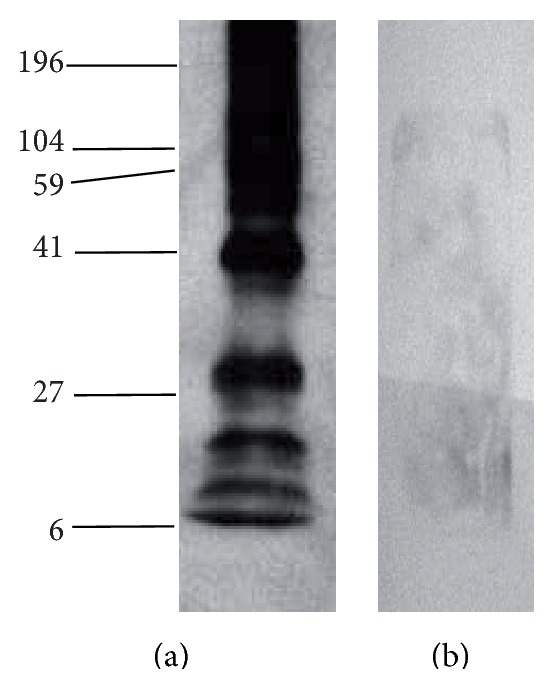
Immunoreactivity against isolated antigens. Representative western blot analysis of* PyL* antigens obtained through immunoaffinity chromatography with immobilized IgGs from malaria surviving ICR mice. (a) shows the specificity of immune sera from ICR mice against purified antigens followed by the incubation with the secondary antibody anti-mouse IgG/HRP linked Fab and (b) was incubated only with the secondary antibody. Lanes were loaded with 10 *μ*g of protein.

As shown in Figure 1(a), a wide range of molecular weight proteins between 22 and 250 kDa were detected by the immune sera, demonstrating that the isolated immune affinity antigens have functional binding and recognition. Moreover, analysis of the antigens with only anti-mouse IgG/HRP linked F(ab) did not show any signal in the membranes (Figure 1(b)), establishing that either complete or fraction IgGs did not coelute in the flow-through during purification. This initial screening of the immunoaffinity isolated antigens that were subsequently used for immunization demonstrated their richness in multiple native IgGs recognition and consequently their potential use for vaccination purposes.

### 3.2. Blood-Stage Antigens Required Adjuvant to Elicit Immune Response

**Figure 2:**
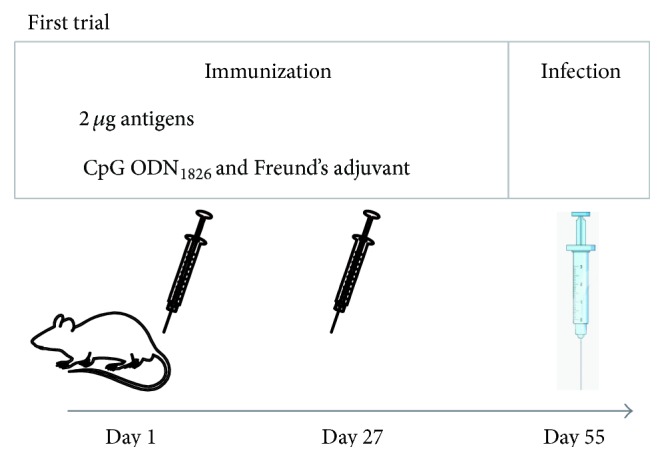
Experimental design of immunization with purified antigens and different adjuvants. IgGs, from naturally surviving ICR mice to* PyL* infection, were purified and used to isolate blood-stage parasite antigens. BALB/c mice divided into 6 groups were then inoculated with 2 *μ*g of purified antigens using CpG ODN_1826_ and/or Freund's adjuvant systems or with proper controls on days 1 and 27 and infected with 2 × 10^7^ *PyL-*RBCs on day 55.

**Figure 3:**
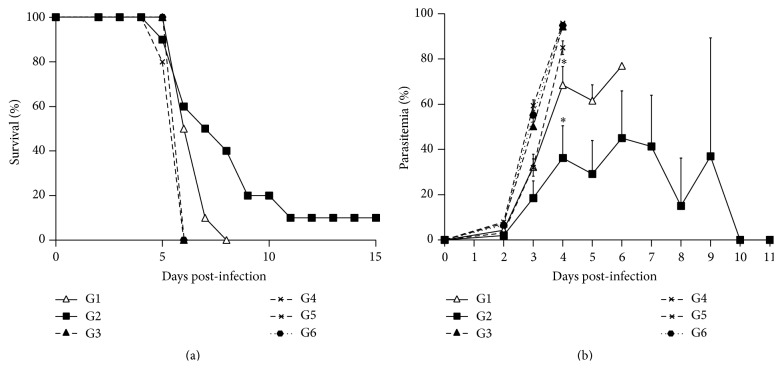
Course of infection after vaccination with different adjuvants in BALB/c mice. Mice were divided into 6 groups (*n* = 10 each) to be immunized on days 1 and 27. Group (G) 1 with 2 *μ*g of purified antigens and CpG ODN_1826_ plus IFA, G2 with 2 *μ*g antigens plus CFA, G3 with 2 *μ*g of antigens, G4 with PBS and CpG ODN_1826_ plus IFA, G5 with PBS and CFA, and G6 with PBS. On day 27 CFA was replaced with IFA. At day 55, all mice were challenged with 2 × 10^7^ *PyL-*RBCs. Percentages of (a) survival and (b) parasitemia are shown as mean (± SEM). ^*∗*^ *P* < 0.05 comparing to controls with antigens or same adjuvant alone or PBS (G2 versus G3, G5, and G6; G1 versus G3, G4, and G6).

*PyL* antigens purified with IgGs antibodies from ICR resistant-mouse sera were used as a vaccine to determine their capacity to induce protection in a uniformly lethal model of malaria as BALB/c mice infected with* PyL*. Due to the difficulties in developing a vaccine formulation, in a first trial the antigens were explored with different adjuvants to test their stimulatory capacity. Mice were challenged with only 2 *μ*g of purified antigens accompanied by CpG ODN_1826_, IFA, or CFA on days 1 and 27 and infected on day 55 (Figure 2). The number of days of life of mice from groups immunized with 2 *μ*g of purified antigens together with any of the tested adjuvant systems (groups 1 and 2) was significantly higher than their control groups 3, 4, and 6; and 3, 5, and 6, respectively (*P* < 0.05) (Figure 3(a)), suggesting that the presence of adjuvants is necessary for the acquisition of some degree of protection. Apart from increasing survival rates, significant lower parasitemia was also observed on day 4  post-infection (pi) between groups 1 and 2 and their own controls (Figure 3(b)).

Since adjuvant CpG is especially effective when small amounts of antigen are applied [26], it was considered potentially suitable for this type of vaccination, particularly due to the difficulty in obtaining large amounts of highly purified antigens. However, although a slight delay in mortality was observed, this adjuvant failed to increase protection against lethal challenging infection with* PyL* (mouse group 1) showing that the choice of an appropriate adjuvant system is critical in the immunization effectiveness with the purified antigens in our mouse model. The most effective adjuvant in our experiments was Freund's. Mice treated with antigens and CFA appeared to be partially protected, as death was delayed for about 2 days, and one mouse survived the challenge.

### 3.3. Immunization Elicits Progressive IgG Response to Multiple Blood-Stage Parasite Antigens

**Figure 4:**
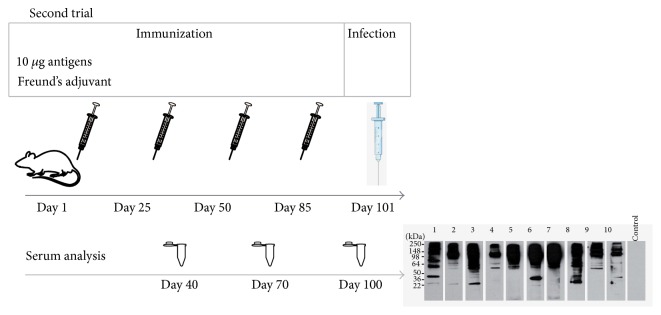
Experimental design of immunization with purified antigens and Freund's adjuvant. Blood-stage parasite antigens were isolated by using IgGs from surviving ICR mice to* PyL* infection and were inoculated together with Freund's adjuvant in BALB/c mice on days 1, 25, 50, and 85. Two weeks later mice were infected with 2 × 10^7^ *PyL*-RBCs. Mouse sera obtained on days 40, 70, and 100 were subjected to immunoblot analysis.

The results obtained using 2 *μ*g of purified antigens for immunization (Figure 3) suggested that the vaccine dose used could be limiting to observe a consistent response. Hence, we increased the amount of immunoaffinity-purified antigens for vaccination to 10 *μ*g, doubled to 4 inoculations along 12 weeks, and tested subcutaneous (s.c.) route of inoculation in order to improve immunogenicity [27]. Thus, BALB/c mice were inoculated with antigens using the Freund's adjuvant system on days 1, 25, 50, and 85 (Figure 4). Our objective was to induce B lymphocytes to synthesize antibodies as they are crucial components of the protective immune response against malaria in human and animal models [28, 29]. Consequently, to ascertain the possible humoral response developed by mice we conducted a time-course IgG immunoblot analysis prior to infection at days 40, 70, and 100 using total* PyL* protein extracts.

**Figure 5:**
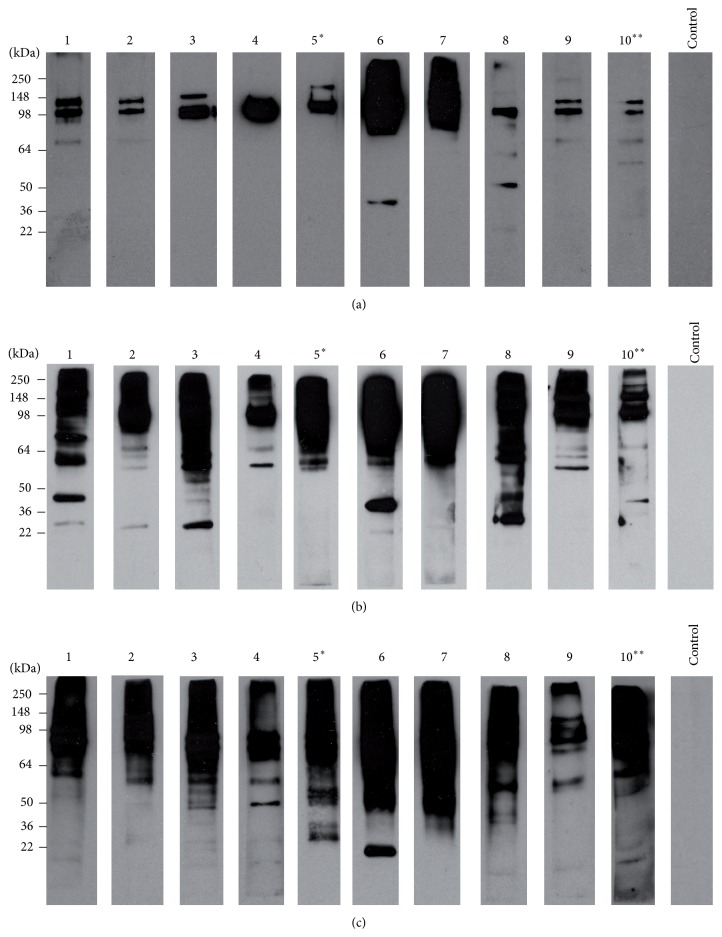
Humoral response of BALB/c mice after immunization with purified* PyL* antigens and Freund's adjuvant. The reactivity of sera obtained from mice previously immunized with highly affinity-purified antigens plus Freund's adjuvant (*n* = 10) was assayed on immunoblots of total* P. yoelii* blood-stage antigen (10 *μ*g/lane) at days 40 (a), 70 (b) and 100 (c). A representative nonvaccinated mouse serum (Control) served as the negative control at corresponding day. Two weeks after last immunization mice were infected with* PyL* and mouse number 5^*∗*^ survived the infection and mouse number 10^*∗∗*^ lived until day 17 pi. Antigen-specific antibodies were detected with anti-mouse IgG/HRP linked F(ab).

A progressive increase of the amount of IgGs and the range of parasite Ags recognized was observed along the vaccination period (Figure 5). At day 40, vaccinated mice sera showed a strong recognition of high molecular weight antigens ranging between 250 and 95 kDa (Figure 5(a)). Moreover, both repertoire and intensity recognition increased at day 70 and subsequently at day 100, when the maximum was observed (Figures 5(b) and 5(c)). From inspection of individual serum reactivity it became apparent that the later IgGs to show up were those recognizing small molecular weight antigens, particularly in the range 64–36 kDa, in which certain degree of heterogeneity was observed between individual mice. Nevertheless, there was no evidence in the individual western blot patterns that could differentiate surviving from nonsurviving mice (Figure 5(c)).

### 3.4. Immunization Elicits Partial Protection against Lethal Blood-Stage Malaria Infection

**Figure 6:**
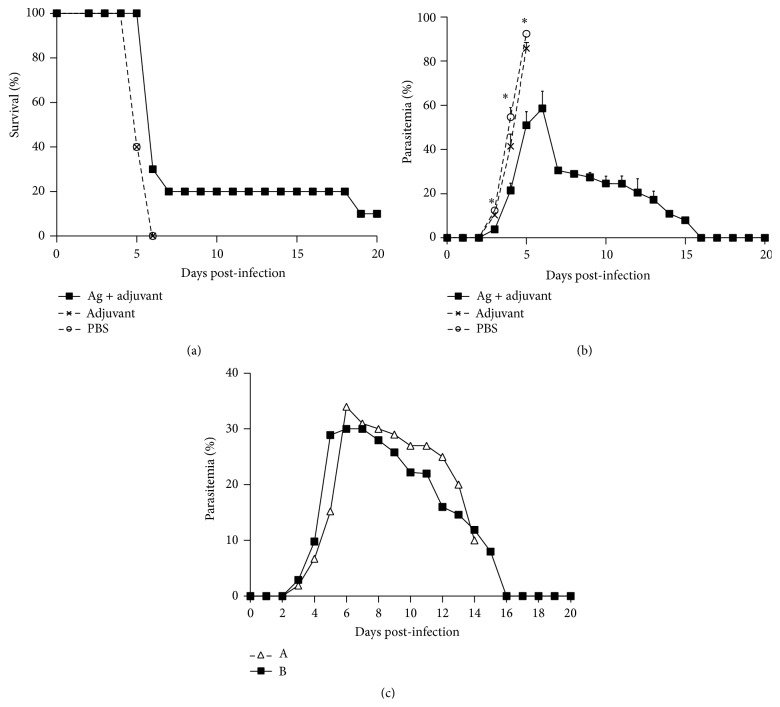
Course of infection in BALB/c mice after antigen and Freund's adjuvant vaccination. Groups of BALB/c mice were immunized with 10 *μ*g of purified antigens formulated with Freund's adjuvant (Ag + adjuvant, *n* = 10), Freund's adjuvant plus PBS (Adjuvant, *n* = 10), or PBS alone (PBS, *n* = 5). Two weeks after the fourth immunization, mice were challenged with 2 × 10^7^ *PyL*-RBCs. Percentages of (a) survival and (b) parasitemia from a total of 500 RBCs are shown as mean (± SEM). (c) Parasitemia course of mice with longer surviving rates after vaccination: mouse which survived around two weeks (Δ) or completely survived (■). Data is shown as mean (± SEM). ^*∗*^ *P* < 0.05 comparing group “Ag + adjuvant” to both controls.

After confirming the immune stimulatory capacity of our antigens with Freund's adjuvant, mice were challenged two weeks after the last immunization to determine their state of immunity to the live parasite. Vaccinated mice survived more days than untreated or adjuvant-treated mice (Figure 6(a)) (*P* < 0.05). In addition, one vaccinated mouse survived up to day 18 pi and another fully survived after clearing parasitemia (Figures 6(a) and 6(b)). Interestingly, parasitemia on these two mice reached a maximum of 30.5% on day 7 pi, much lower than the maximum of 92.4% by day 5 pi reached in control mice, and progressively declined during the following days (Figure 6(c)). At day 17 pi, one out of two vaccinated mice died with a status of severe anaemia, while the surviving mouse cleared all parasites in blood as observed by microscopy inspection.

## 4. Discussion

The incomplete immune response to malaria together with the lack of a licensed vaccine and the spread of drug resistant parasites hinder malaria control and turn malaria disease into a major public health problem [30]. Numerous vaccine strategies are being researched in the malaria field and are mainly based on three different antigens separately used: circumsporozoite protein (CSP), merozoite surface protein (MSP), and apical membrane antigen 1 (AMA-1). However, these strategies do not emulate the naturally acquired immunity against malaria which is based on years of repetitive infections allowing contact with a large range of different antigens [5, 7, 31]. Actually, immunizations with a broad spectrum of antigens from different parasite stages are considered to counteract the parasite's immune evasion improving protection [8]. According to this concept, experimental vaccination with whole parasites through irradiation [32] or genetically [33] attenuated* Plasmodium* sporozoites or with parasites in hepatic or blood-stages under treatment with different drugs [13, 14, 34] is providing excellent results of malaria protection as they can more easily fight against the antigenic polymorphism of the parasite. Thus, in the present work, we aimed to produce an immunogenic combination based on* P. yoelii* blood-stage antigens isolated with immobilized affinity-purified antibodies from malaria-resistant mice. During blood-stage malaria infection, parasite-specific antibodies have been shown to play a key role in controlling parasitemia [28, 35]. However, malaria infection also gives rise to strongly elevated blood concentrations of non-malaria-specific immunoglobulin [36] and IgG responses specific for* P. falciparum* antigens are often unexpectedly short-lived and fail to consistently boost upon reinfection [37]. For that reason, the research of protective antigens is still difficult and here we proposed to use the total* Plasmodium*-specific IgGs produced in response to malaria reinfection in ICR immune mice to isolate total antigens in which protective antigens will also be present. Although resistance to malaria can be generated through antimalarial treatment in mice [38], drugs can alter the natural mechanisms of immune protection. Consequently, to test the protective capacity of* PyL* antigens we chose to use antibodies produced under a naturally developed immune response in the ICR malaria model in which a 20% of mice naturally develop immunity to the infection [20].

We have previously demonstrated the protective role of antibodies against the infection through the passive transfer of pooled sera from resistant ICR mice containing 150–200 *μ*g of total IgG to naïve BALB/c mice, 2 hours after a challenge with 2 × 10^7^ *PyL* iRBCs, being able to cure 40% of transferred mice [20]. BALB/c mice are uniformly susceptible to fatal malaria infection with* PyL* so their use for vaccine and drug preclinical trials is consolidated [24].

This procedure allows us to previously identify well-known and new malaria antigens including protein disulfide isomerase (PDI), the HSPA5 member of the heat shock protein-70 family (also called binding immunoglobulin protein BiP), the aspartic proteinase plasmepsin, and the eukaryotic translation initiation factor 3 (IF3) as antigenic proteins [21]. In addition to these proteins, other unidentified bands are also detected on 1D-electrophoresis within the immune-affinity purified proteins, suggesting that the partial protection of BALB/c mice against the lethal infection dose is not only due to the above indicated identified proteins. Nonetheless the potential role of those four highly prevalent antigens to immunize the surviving mice may contribute to the increase of the components of a more effective blood-stage malaria vaccine. Immune responses to malaria HSPs have been repeatedly demonstrated in patients with malaria [39]. Further, several reports characterizing malarial HSPs indicated that this protein class might play important roles in the life cycle of the parasites [40]. The ability of HSP 70 to regulate parasite actin polymerization during invasion of RBCs suggests that HSP 70 is correlated with infectivity of malaria parasites [40, 41]. The remaining three identified plasmodial immunogenic antigens, for which their functionality in blood-stage malaria has been well defined elsewhere [21], are similarly worth being explored as candidates to raise antibodies during* in vivo* vaccination screens with purified antigens.

Among the criteria for selecting a vaccine formulation, the antigenic components are crucial to later investigate adjuvants, route of delivery, potential adverse effects, and stability. Given the large variety of responses that the same antigens can generate with different adjuvants [42–44], we chose to compare the effect of two different adjuvants to test the* in vivo* protective immunity with our set of* P. yoelii* purified antigens. Results obtained using 2 *μ*g of purified antigens as potential immunogenic mixture showed some degree of protective response, which was dependent on the adjuvants used.

It has been reported that CPG ODNs binding to TLR9 receptor results in the production of proinflammatory cytokines and chemokines stimulating the recruitment of additional inflammatory mediators. However, our isolated antigens plus CpG ODN adjuvant did not confer protection against the malaria infection in mice. Actually, immunization with CpG ODN has shown a wide variety of results in infectious disease trials [45]. Factors as conjugation rate of protein and ODN, infection dose, and administration route play a role in its effectiveness as adjuvant [46–48].

On the other hand, Freund's adjuvant decreased parasitemia and increased survival in the vaccinated mice. It has been reported that early immune response caused by IFA/CFA includes rapid uptake of adjuvant components by dendritic cells, enhanced phagocytosis, secretion of cytokines by mononuclear phagocytes as well as transient activation and proliferation of CD4+ lymphocytes [49] in addition to antibody production against denatured epitopes of proteins [50]. Given that Freund's adjuvant was more effective in our experiments we chose this adjuvant in the formulation of the following vaccine trial.

In the second trial, the humoral response developed by BALB/c mice after the four vaccination challenges was characterized by immunoblot which confirmed the immunogenicity of our isolated antigens. A large variety of* PyL* specific IgGs were developed against a wide range of parasite proteins, similar to the build-up of the humoral response observed in partially immunized humans [51]. The key role of B cells in controlling malaria infections has been clearly revealed in rodent models lacking these cells, which are not able to eliminate* P. yoelii* [35] and* P. chabaudi* infections [52]. Moreover, the protective role of Abs against malaria infection is supported by the transfer of immune serum into infected nonimmune humans as an efficient treatment strategy [28] that is also efficient in mouse models [29]. Specific antimalarial Abs might protect by several attributes, including merozoite invasion blocking, cooperation with monocytes, NKs or DCs; or by inhibiting iRBCs cytoadherence [36].

Infection of our vaccinated mice showed that all individuals prolonged their survival rate but only a small fraction was really protected against the lethal infection. Further experiments with lower infective doses could permit a progressive encounter of immune system with the parasite and influence Th1/Th2 activity [53]. Besides, the inoculation of a higher quantity of antigen could probably improve the percentage of protection. Immune responses in endemic infections in humans can be partially protective, allowing the individual to tolerate significant parasite densities without overt disease [54], but the mechanisms of immune protection are poorly understood [1, 55]. In fact, we were not able to identify differential antibody production between protected and unprotected individuals which could be the reason of the low duration of the protection, as it has also been described following the administration of the RTS, S/AS01, the most advanced vaccine candidate in humans [56–58].

## 5. Conclusions

In conclusion, immunoaffinity-purified antigens using IgGs from protected individuals deserve further research, either by fractionation of some of the isolated set or by complementation with additional components, to improve efficacy of a potential multiantigen blood-stage malaria vaccine.
